# Pathologic Complete Response to Neoadjuvant Radiation Therapy in a Primary Pancreaticoduodenal High-Grade Sarcoma

**DOI:** 10.7759/cureus.91686

**Published:** 2025-09-05

**Authors:** Jenna R Rocchetti, Jeffrey M Farma, Alessandro Bombonati, Mehri Mollaee, Jeremy Price

**Affiliations:** 1 Radiation Oncology, Fox Chase Cancer Center, Philadelphia, USA; 2 Surgical Oncology, Fox Chase Cancer Center, Philadelphia, USA; 3 Clinical Pathology, Temple University Hospital, Philadelphia, USA; 4 Radiation Oncology, Fox Chase Cancer Center, Temple University Hospital, Philadelphia, USA

**Keywords:** high-grade intraabdominal sarcoma, pancreaticoduodenal sarcoma, pathologic complete response, radiation therapy, soft tissue sarcoma

## Abstract

High-grade intra-abdominal sarcomas are a rare soft tissue malignancy with a high propensity to present in a locally advanced fashion. The role of radiation therapy (RT) in the management of intra-abdominal sarcomas is relatively under-reported, in contrast to its use in retroperitoneal or extremity sarcomas. We thereby present a case of a 63-year-old female presenting with abdominal pain, gastrointestinal (GI) bleeding, and weight loss, found to have a large, high-grade intra-abdominal pancreaticoduodenal sarcoma. Given ongoing bleeding, absence of actionable mutations, and surgical risk, she was treated with neoadjuvant RT (50 Gray in 25 fractions) using image-guided volumetric-modulated arc therapy. The patient tolerated treatment well with rapid and significant tumor regression, allowing for subsequent pancreaticoduodenectomy. Pathology revealed a complete pathologic response (ypT0N0M0) with no residual viable tumor. At 2.5 years post-treatment, she remains disease-free. This case highlights the potential role of neoadjuvant RT in achieving local control and facilitating curative resection in patients with high-grade intra-abdominal sarcomas. While evidence is limited, especially for visceral intra-abdominal sites, this report supports further investigation into RT as a component of multimodal therapy in select cases.

## Introduction

Soft tissue sarcomas are malignant tumors of mesenchymal origin, with intra-abdominal soft tissue sarcomas comprising only 0.1% of adult malignant tumors [1]. Although they are seemingly rare, high-grade intra-abdominal sarcomas are prone to progression and recurrence, so early detection and aggressive management are crucial for improving outcomes [2]. The severity of the disease may be because these tumors are often confined within the abdomen, causing them to go undetected for an extended period of time, leading to significant tumor growth and a greater risk of local invasion [3]. The spectrum of etiologies of intra-abdominal sarcomas is broad, including diseases such as gastrointestinal stromal tumors (GISTs), retroperitoneal sarcomas, and visceral intra-abdominal sarcomas. 

The typical management for intra-abdominal sarcomas is surgery [4]. Surgical removal of the tumor requires careful attention to not rupture the pseudocapsule, as this can lead to tumor spillage and increased risk of peritoneal recurrence [4]. Unfortunately, less than 50% of patients have localized disease at diagnosis; therefore, when disease is extensive or patients are deemed unfit for surgery, systemic therapies or radiation are considered.

The role of radiation therapy (RT) for sarcomas of the visceral abdomen in these scenarios is unclear, with sparse evidence supporting its use, which may be due to the rarity of the disease and the limited dose tolerance of the GI organs [5]. This contrasts with the more robust literature describing retroperitoneal sarcomas. However, advancements in radiation modalities such as daily image-guided radiation therapy (IGRT) and intensity-modulated radiation therapy (IMRT) have led to promising results for achieving control of high-grade intra-abdominal sarcomas [6]. Herein, we present a case of a primary intra-abdominal high-grade pancreaticoduodenal sarcoma. Neoadjuvant RT was the consensus recommendation from our multidisciplinary sarcoma tumor board to address acute symptoms and to downstage the tumor prior to radical resection, ideally decreasing the risk of local recurrence. RT led to a pathologic complete response with no residual viable tumor found after pancreaticoduodenectomy. This case report was de-identified and received a waiver from our institutional review board before publishing.

## Case presentation

The patient is a 63-year-old female who presented with an acute-on-chronic onset of fevers and abdominal pain for three months. She also reported anorexia, diminished appetite, and a 20-pound weight loss during that time. After initial presentation to her primary care physician, an abdominal ultrasound was ordered, which demonstrated an irregular, hypoechoic mass measuring 5.8x4.7x5.5 cm of duodenal versus pancreatic origin. No distinct ductal dilation was seen, and there was no evidence of gastric outlet obstruction.

**Figure 1:**
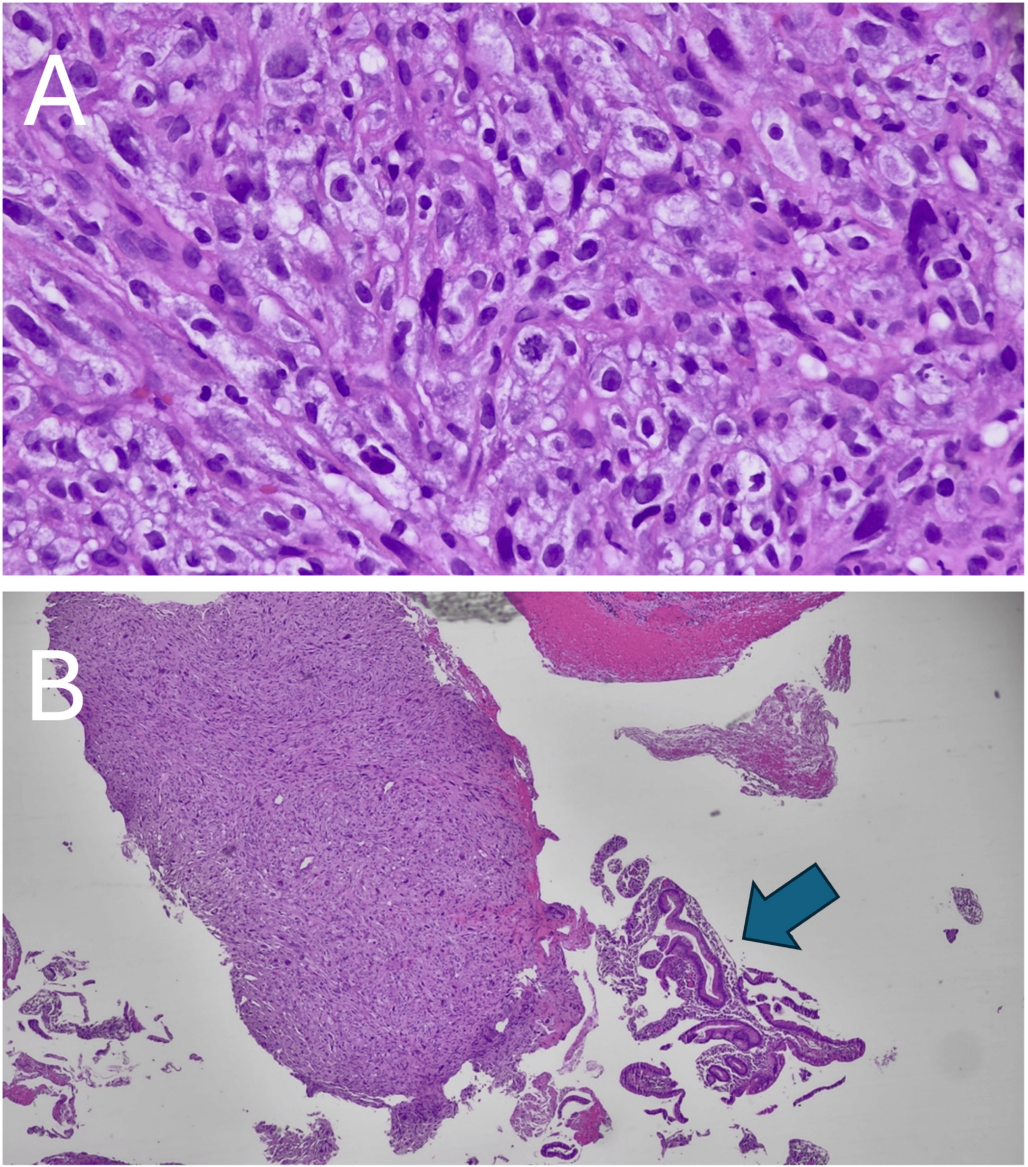
Histopathology of intra-abdominal pancreaticoduodenal sarcoma. (A) High-grade sarcoma (400×) showing pleomorphic and spindle-shaped tumor cells; (B) High-grade sarcoma (100×) showing the overall tumor with strips of overlying benign duodenal mucosa (blue arrow).

**Figure 2:**
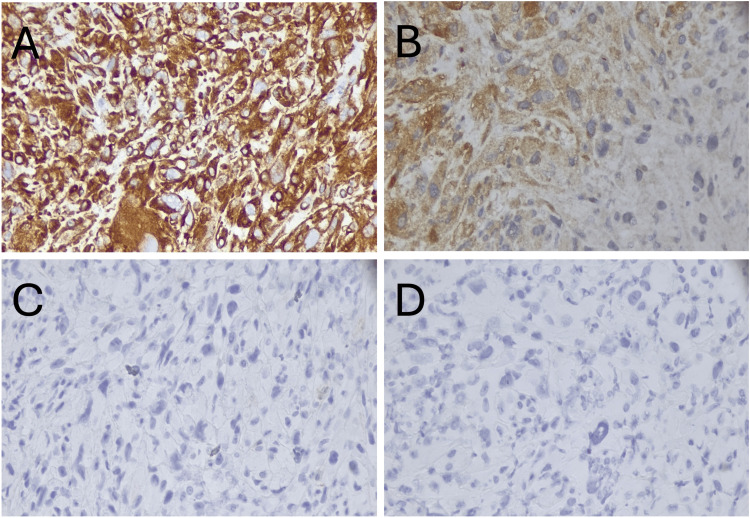
Intra-abdominal pancreaticoduodenal sarcoma immunostaining. Pathologic immunohistochemistry studies, all shown at 400×: (A) Vimentin; (B) Desmin; (C) S100; (D) DOG-1; (E) CAM5.2.

A small intestine/duodenal fine-needle aspiration biopsy was performed during endoscopic retrograde cholangiopancreatography, and pathology showed a high-grade undifferentiated malignancy (Figure 1), negative for DOG-1, SMA, S100, AE1/AE3, CK7, CK20, CK903, CAM5.2, p63, CD34, PAX-8, and calretinin. The tumor cells were positive for vimentin, CD68, and focally for desmin (Figure 2). Diffuse positivity for vimentin and focal positivity for desmin support the tumor’s mesenchymal origin and smooth muscle differentiation, respectively. Negativity for S100, DOG-1, and CAM5.2 helps rule out melanoma, GIST, and carcinoma, respectively. The tumor was also negative for cKIT and PDGFR. After intradepartmental review, this was felt to be most consistent with a high-grade intra-abdominal sarcoma with smooth muscle differentiation.

**Figure 3:**
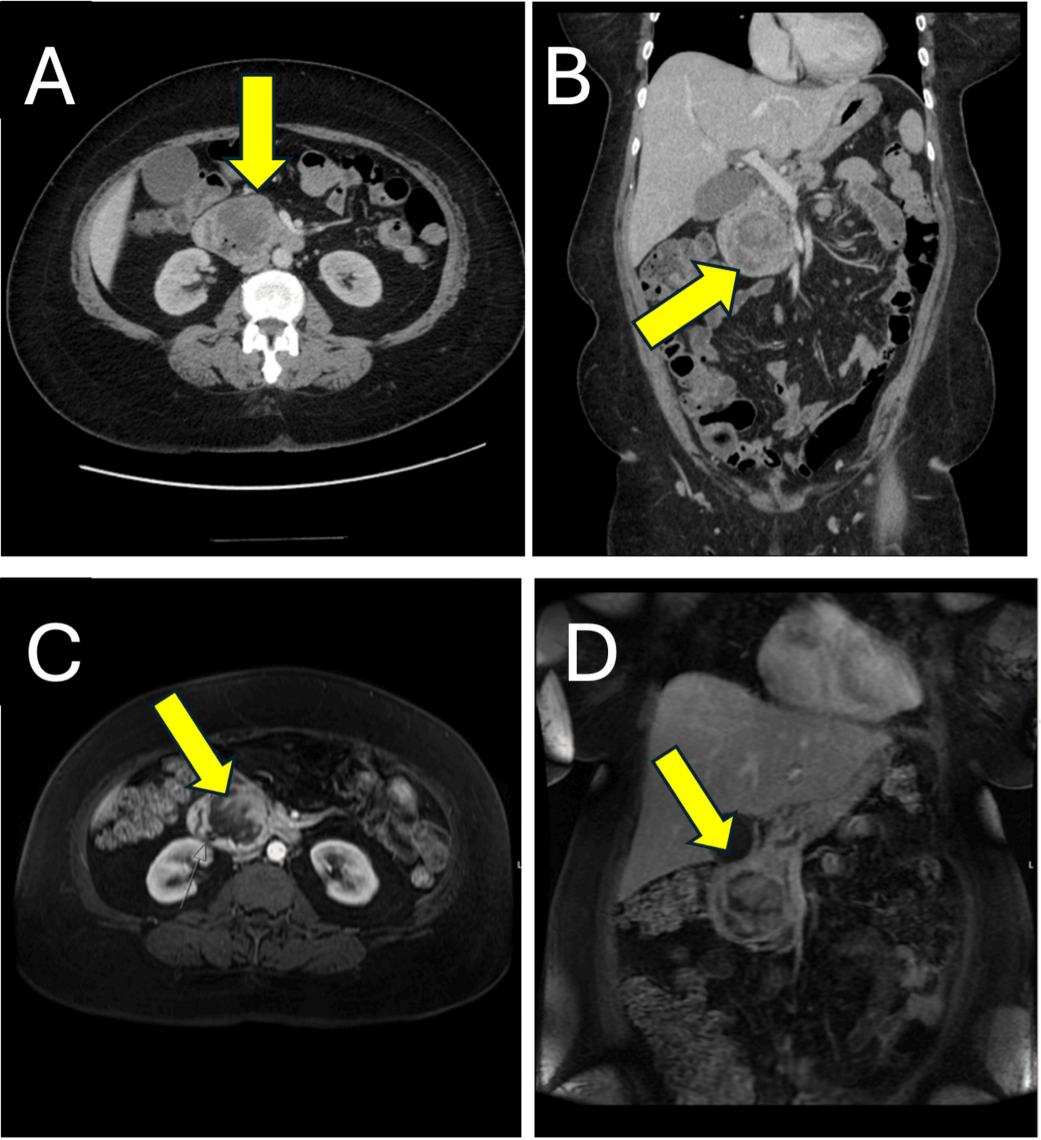
CT of the chest, abdomen, and pelvis with contrast and MRI of the primary pancreaticoduodenal soft tissue mass. (A) CT axial view, (B) CT coronal view, (C) MRI axial view, (D) MRI coronal view, with the yellow arrow pointing to an expansile mass (5.4×5.1 cm) in the region of the duodenum and pancreatic head, with no evidence of metastatic disease. CT, computed tomography; MRI, magnetic resonance imaging

A computed tomography (CT) scan of the chest, abdomen, and pelvis with contrast was then performed, which showed no evidence of metastatic disease but revealed an expansile, heterogeneous, hypoattenuating soft tissue mass in the region of the proximal duodenum and pancreatic head. There was a mass effect on the common bile duct and distal pancreatic duct, with mild associated intrahepatic biliary duct dilation, pancreatic duct dilation, and mild atrophy of the distal body and tail of the pancreas. Additional magnetic resonance imaging (MRI) of the abdomen with and without contrast redemonstrated the primary pancreaticoduodenal mass, measuring 5.4×5.1 cm, with partial encasement of the duodenum and cystic changes/necrosis. This demonstrates a “claw sign” involving both the pancreas and duodenum (Figure 3).

At this point, the patient began to experience ongoing and sustained GI bleeding, with hemoglobin falling below 7 g/dL. Repeat imaging showed no acute changes in the primary pancreaticoduodenal mass. A biliary stent was therefore placed, and her case was discussed at our multidisciplinary sarcoma tumor board. In part due to her severe anemia and lack of actionable mutations, neoadjuvant chemotherapy was deferred in favor of neoadjuvant RT, following a paradigm similar to that of borderline resectable pancreatic adenocarcinomas [7]. Given the ongoing bleeding and concern for surgical risk, the patient was recommended to receive neoadjuvant RT as a temporizing measure to stop her bleeding and treat the sarcoma. The patient was clinically assessed as stage IIIA (cT2N0M0G3).

**Figure 4:**
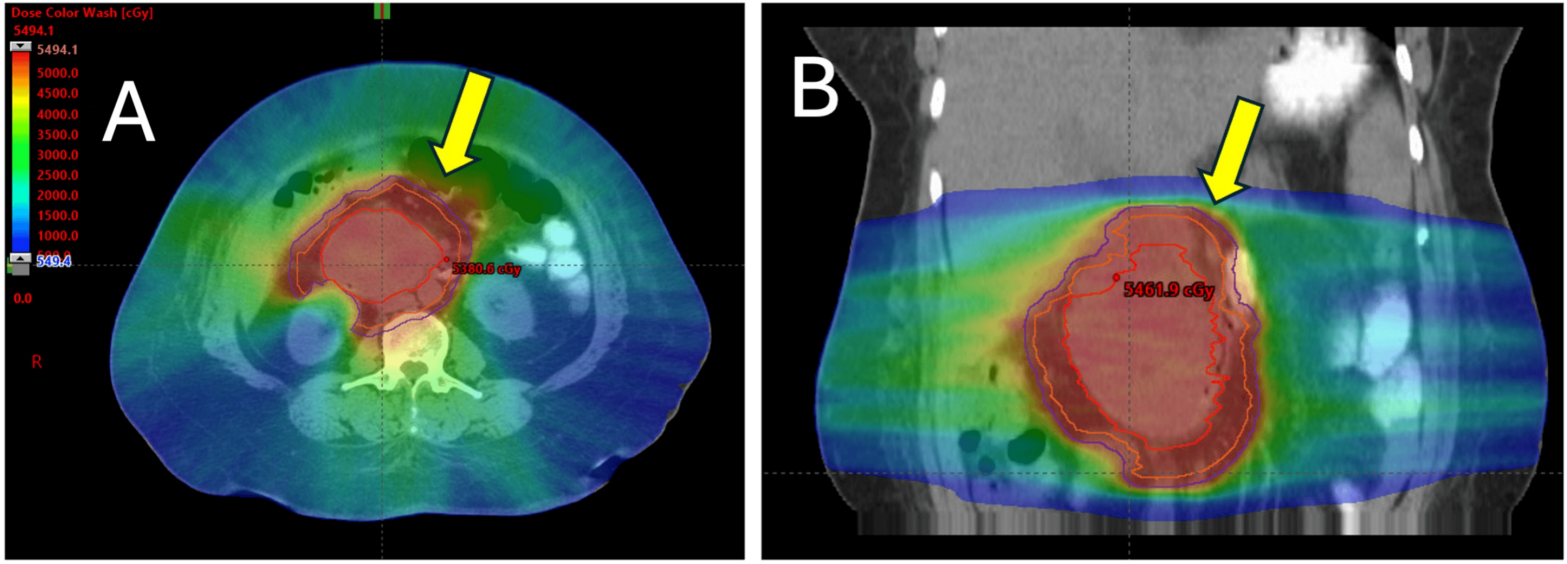
RT simulation and plan. The prescription dose was 50 Gray in 25 fractions, delivered via VMAT. (A) Axial view and (B) coronal view, with the yellow arrow pointing to the mass. GTV=red, ITV=orange, PTV=blue. GTV, gross tumor volume; ITV, internal target volume; PTV, planning target volume; VMAT, volumetric modulated arc therapy

RT was pursued with a neoadjuvant dose prescription of 50 Gray (Gy) in 25 fractions, administered once daily. The plan was delivered by volumetric-modulated arc therapy (VMAT) with two coplanar, six medium-voltage (MV) arcs. For planning, the patient was immobilized in a vacuum-lock cushion and received a 4D simulation to account for intra-abdominal motion. She was instructed to avoid oral intake for three hours prior to simulation and daily RT. The internal gross target volume (IGTV) encompassed the gross tumor on the simulation 4D scan and fused diagnostic CT/MRI imaging. The 4D scan was then used to generate an internal target volume (ITV) in lieu of a clinical target volume (CTV) by applying a 5 mm margin to the IGTV, cropped to exclude adjacent organs at risk (OAR), and respecting anatomic boundaries. A planning target volume (PTV) was generated by adding an additional 5 mm expansion of the ITV (Figure 4).

**Figure 5:**
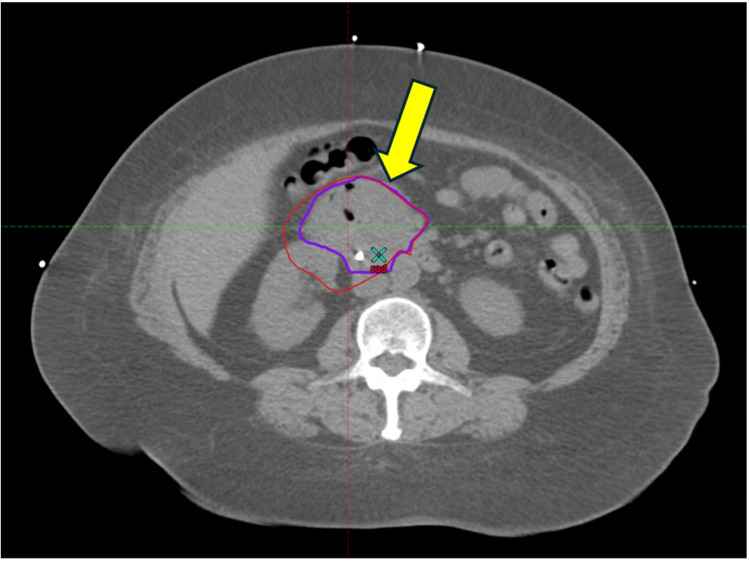
Resimulated RT plan after 32 Gray. Axial view with the yellow arrow pointing to the pancreaticoduodenal mass, which decreased in size after initiation of therapy, necessitating re-simulation. Old IGTV=red, new IGTV=purple RT, radiation therapy; IGTV, internal gross target volume

After initiation of therapy, the patient tolerated treatment well overall and was noted to have a rapid interval decrease in the size of the pancreaticoduodenal tumor. The patient was therefore re-simulated after 32 Gy, as the right kidney fell into the PTV due to tumor volume reduction, and was placed on a temporary treatment break (Figure 5). The maximum right kidney dose was reduced from a planned 52.6 Gy to 51.4 Gy. The mean kidney dose fell from a planned 16.5 Gy to 14.1 Gy. The actual delivered dose to the right kidney without a replan would have been substantially higher, with maximum and mean doses of 55.5 Gy and 22.1 Gy, respectively.

**Figure 6:**
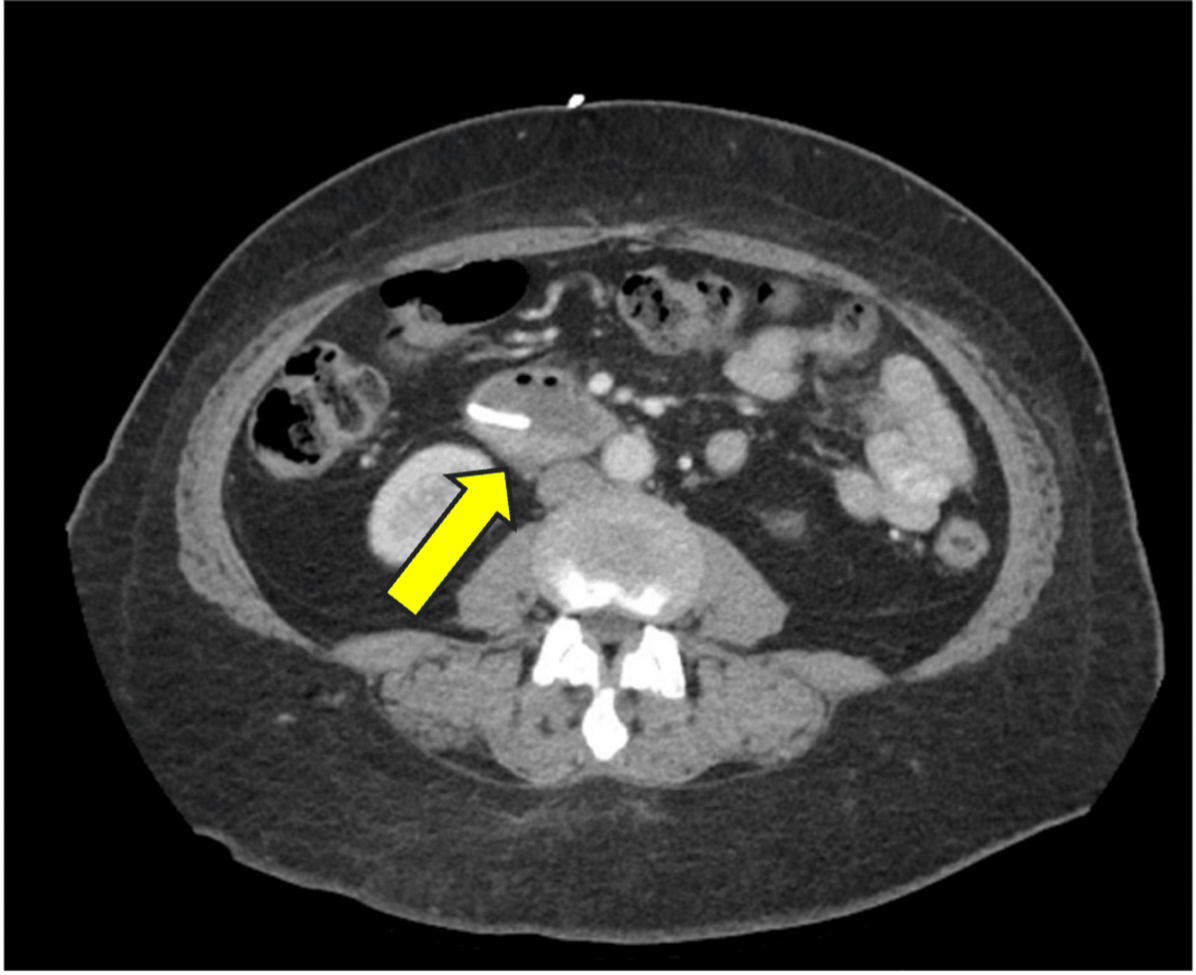
Radiographic response of the primary tumor one month after RT. Axial view with the yellow arrow pointing to the pancreaticoduodenal mass. RT, radiation therapy

The patient completed radiation five weeks after initiation, with minimal treatment interruption due to re-simulation and replanning. One month post-RT, a restaging CT scan of the abdomen and pelvis was performed, showing minimal residual thickening of the duodenal wall (Figure 6).

Eight weeks after completion of RT, the patient underwent a pancreaticoduodenectomy. Final pathology revealed no tumor, compatible with a complete response to radiation (ypT0N0M0). Post-treatment surveillance included CT of the abdomen and pelvis every three months, along with a history and physical examination. The patient remains disease-free 2.5 years after completion of RT. The postoperative course was complicated by delayed gastric emptying and an enterocutaneous fistula, requiring multiple readmissions and eventual percutaneous gastrostomy tube placement. The fistula eventually healed, and her gastrostomy tube was subsequently removed.

## Discussion

Here we report a case of a high-grade intra-abdominal pancreaticoduodenal sarcoma. Neoadjuvant RT was administered and led to a complete response following pancreaticoduodenectomy. There are a limited number of studies addressing the role of RT in the management of high-grade intra-abdominal sarcomas.

Diagnosing intra-abdominal sarcomas presents significant challenges due to the wide variability in patient presentations. Although not an absolute method for confirming the diagnosis, immunohistochemical staining of biopsies can serve as a valuable tool in tumor classification. In the case of our patient, the c-Kit-negative status, coupled with the absence of DOG1 expression, further helped to rule out GIST as a potential diagnosis. Additionally, the tumor was desmin positive, supporting smooth muscle differentiation. Notably, approximately 30% of c-Kit-negative tumors exhibit desmin positivity [8].

Our patient’s intra-abdominal malignancy was effectively controlled with neoadjuvant RT; however, some intrabdominal tumors may progress to an extent that only palliative care remains a viable option. Zahra et al. reported a case of a 55-year-old female with a primary intra-abdominal renal synovial sarcoma, whose disease progressed following radical nephrectomy, necessitating chemotherapy and RT with palliative intent [2]. This underscores the need for ongoing research to refine management strategies for intra-abdominal sarcomas, given their aggressive nature and high recurrence rates.

Another exceedingly rare type of intra-abdominal sarcoma includes primary duodenal liposarcomas, which must be distinguished from more common retroperitoneal liposarcomas that invade the duodenum [9]. Whitham et al. describe a 59-year-old woman with an intra-abdominal primary duodenal dedifferentiated liposarcoma who remained disease-free 16 months after proximal duodenectomy and distal gastrectomy with Roux-en-Y reconstruction. Unlike our case, she did not require pancreaticoduodenectomy because the mass did not extend to the second portion of the duodenum [10]. The tumor stained positive for MDM2 and CDK4, which are specific for dedifferentiated liposarcomas [10,11]. Similar to our patient, c-Kit and DOG1 immunohistochemical stains were negative, but vimentin was positive. Alarmingly, dedifferentiated liposarcomas have local recurrence in 40% of cases and metastasis in 17%, emphasizing the importance of long-term follow-up [10]. Given their aggressiveness, differentiating dedifferentiated liposarcomas from other GI tumors, especially retroperitoneal tumors, is crucial for determining prognosis.

While data on intra-abdominal sarcomas are comparatively rare, there is more widespread evidence examining the use of RT in the management of retroperitoneal sarcomas. Retroperitoneal sarcomas represent approximately 15% of all soft tissue sarcomas [12], and unfortunately, local recurrence accounts for nearly 75% of retroperitoneal sarcoma-related deaths [13]. Similar to the presented intra-abdominal sarcoma, retroperitoneal sarcomas are difficult to manage because they often have a delayed presentation involving critical organs and structures, making surgery challenging [14]. Even with optimal surgery, rates of local recurrence remain 25%-50% [13]. Although surgery remains the cornerstone of treatment, it requires major planning and preparation to be successful, with a risk of significant blood loss [14,15].

As with intra-abdominal sarcomas, the role of curative RT in the management of primary localized retroperitoneal sarcomas is still highly debated, with some studies showing a local control benefit, while others conclude that RT does not provide a meaningful advantage for these patients [14]. Furthermore, the timing of RT in retroperitoneal sarcomas has been studied. The National Cancer Institute conducted a trial in 1993 comparing intraoperative RT to adjuvant RT. Although they found similar survival outcomes, there were fewer local recurrences and complications in the intraoperative radiation group [16].

Most recently, the randomized phase 3 STRASS-1 trial explored neoadjuvant RT (50 Gy) in retroperitoneal sarcomas compared to surgery alone. While there was no improvement in abdominal recurrence-free survival [15] for all patients with the addition of neoadjuvant RT, there was a potential benefit for patients with liposarcomas. This study has been criticized due to its use of the composite endpoint of abdominal recurrence-free survival, which may have overestimated cases of local progression in the preoperative radiotherapy arm, thus calling into question the conclusions of the STRASS-1 study [16]. A relatively new trial, known as the STREXIT trial, further supports that RT is useful and safe for neoadjuvant therapy of sarcomas adjacent to abdominal organs at risk [17]. A similar phase II trial evaluated preoperative high-dose RT (54 Gy) for retroperitoneal liposarcomas, showing long-term oncologic control with a three-year locoregional relapse-free survival rate of 74.3% [18]. However, 6.3% of patients experienced grade 5 radiation toxicities, and 19% developed second cancers, suggesting that 54 Gy may carry significant risks [18]. This phase II trial, along with the STRASS-1 trial, suggests that the ideal indications for RT in patients with retroperitoneal sarcomas are still to be determined.

## Conclusions

This case demonstrates that neoadjuvant RT may be a potent, potentially underutilized tool in the management of high-grade intra-abdominal sarcomas. Following combined neoadjuvant RT and pancreaticoduodenectomy, our patient achieved an early radiographic response, a complete pathologic response at the time of surgery, and durable disease control more than 2.5 years after completion of therapy. Prior literature on RT for intra-abdominal sarcomas has been limited to several case series, and this report further supports the use of RT as an adjunct to surgical and systemic therapy on a patient-specific basis. In select cases, including ours, RT has been shown to provide objective responses and long-term disease control without significant radiation toxicity or complications. These findings therefore support further investigation into the benefits of RT in intra-abdominal sarcoma management.
